# Predator-prey transmission of a gammaherpesvirus from Asian badgers (*Meles leucurus*) to endangered Amur tigers (*Panthera tigris altaica*)

**DOI:** 10.1371/journal.pone.0327463

**Published:** 2025-07-02

**Authors:** Martin Gilbert, Malcolm A.M. Hill, Leah Y.N. Cheung, Patti K. Kiser, Ivan V. Seryodkin, Dale G. Miquelle, John M. Goodrich, Nadezhda Sulikhan, Olga Uphyrkina, Mikhail Goncharuk, Linda Kerley, Ryan M. Troyer

**Affiliations:** 1 Cornell K. Lisa Yang Center for Wildlife Health, Cornell University, Ithaca, New York, United States of America; 2 Wildlife Conservation Society, Bronx, New York, United States of America; 3 Department of Microbiology & Immunology, Western University, London, Ontario, Canada; 4 Department of Pathology and Laboratory Medicine, Western University, London, Ontario, Canada; 5 Pacific Geographical Institute, Far Eastern Branch of the Russian Academy of Sciences, Vladivostok, Russia; 6 Panthera, New York, United States of America; 7 Federal Scientific Center of the East Asia Terrestrial Biodiversity, Far Eastern Branch of the Russian Academy of Sciences, Vladivostok, Russia; 8 Lazovskii State Nature Zapovednik, Primorskii Krai, Russia; 9 Zoological Society of London, London, United Kingdom; Wrocław University of Environmental and Life Sciences: Uniwersytet Przyrodniczy we Wroclawiu, POLAND

## Abstract

We sought to identify herpesviruses in wild Amur tigers (*Panthera tigris altaica*) of the Russian Far East in and near the Sikhote-Alin Biosphere Zapovednik protected area. We used multiple herpesvirus consensus PCRs targeting the glycoprotein B and DNA polymerase genes followed by DNA sequencing to test blood samples collected over a 22-year period. We found identical herpesvirus sequences in 3 of 41 tigers by consensus PCR and 8 of 41 tigers (19.5%) using a virus-specific PCR. Persistent infection was demonstrated in a tiger that remained virus DNA-positive in three blood samples over a 2.5-year period. Surprisingly, the viral DNA sequence present in tigers had 98.8% identity to mustelid gammaherpesvirus 1 (MusGHV1) commonly found in European badgers (*Meles meles*), which do not range to the Russian Far East. We then tested 69 blood samples from 11 other carnivore species collected in this region and found that 81.0% (17/21) of Asian badgers (*Meles leucurus*), but no other species, had MusGHV1 sequences with 99.8–100% identity to those found in tigers. Interaction between Amur tigers and Asian badgers is supported by previous studies demonstrating that badgers are a common prey species for tigers in this region. Taken together, these results are consistent with the interpretation that a strain of MusGHV1 common in Asian badgers was transmitted via predator-prey interactions to Amur tigers. While gammaherpesviruses are generally thought to exhibit strong host species-specificity, our results present an example of cross-species transmission and one of the first examples, to our knowledge, of gammaherpesvirus predator-prey transmission. In addition, we identified novel gammaherpesviruses in sable (*Martes zibellina*), Asiatic black bear (*Ursus thibetanus*), and brown bear (*Ursus arctos*).

## Introduction

The family *Orthoherpesviridae* are a group of double-stranded DNA viruses with large genomes (>100 kb) that infect a wide range of vertebrate species and are classified into three subfamilies: *Alphaherpesvirinae*, *Betaherpesvirinae*, and *Gammaherpesvirinae*. The gammaherpesviruses (GHVs) are a highly diverse group that is further divided into seven genera: *Bossavirus*, *Lymphocryptovirus*, *Macavirus*, *Manticavirus*, *Patagivirus*, *Percavirus*, and *Rhadinovirus*. While GHVs can vary greatly in genetic composition, virus-host interactions, host tropism, and pathologic associations; they share several common features. They incur life-long infection of the host by establishing a latent state in lymphoid cells in which little viral gene expression or virus production occurs [1]. This latent state tends to predominate, but under certain conditions viral gene expression can be reactivated, resulting in a lytic state characterized by active virus production and spread [2]. Some GHVs can induce lymphoproliferative disorders including lymphoma and other cancers [3–5], with occurrence of these diseases typically being more common in immune-compromised hosts [6]. In addition, other pathologies have been associated with less well-studied animal GHVs, such as fibrotic lung disease in horses [7] and potentially other species [8] and negative effects on reproductive fitness of badgers [9]. Thus, GHVs can impact human and animal health but the pathogenic consequences of many animal GHV infections remain largely unknown.

GHVs have been identified in a wide range of animals and most are thought to be highly host species-specific, only capable of infecting one species or a group of very closely related species [10]. However, phylogenetic analysis of extant herpesvirus sequences suggests that host-switching events have occurred numerous times during GHV evolutionary history [11–13]. There are also notable examples of specific cases of GHV cross-species transmission. For instance, malignant catarrhal fever viruses from the *Macavirus* genus of GHVs can transmit from native hosts such as wildebeest and sheep to non-native ruminants such as cattle and deer, resulting in virulent lymphoproliferative disease, but not resulting in continued propagation in the non-native species [3].

Predator-prey interactions are an underappreciated means of pathogen transmission, with predators exposed to a multitude of potential pathogens present in their prey [14]. However, potential GHV transmission via predator-prey interactions has received only sparse research. A thorough study of a primate predator-prey system by Murthy and colleagues demonstrated a lack of GHV transmission from prey primate species to a predator primate species [15]. Our lab and others have recently focused attention on the presence of GHVs from the *Percavirus* genus in host species of the order *Carnivora* [16–20]. Within the percaviruses, there are several examples of potential virus transmission from prey to predator or via other carnivore-carnivore interactions. A GHV found in hyenas clusters phylogenetically with a zebra GHV, suggesting potential historical predator-prey transmission [13]. Similarly, a GHV identified commonly in North American bobcats was also occasionally detected in pumas and Canada lynx [16,17]. Lastly, Tsushima leopard cats were found to carry a GHV present in domestic cats worldwide [21]. Collectively, these findings suggest the potential for GHVs to be transmitted across species to carnivores by predator-prey interactions.

The family *Felidae* includes more than 40 cat species spread throughout the world [22]. Our lab and others have identified unique GHVs in several felid species including domestic cat, lion, bobcat, puma, ocelot, and Canada lynx [13,16–18], suggesting that additional felid species may be infected by other currently-unknown GHVs. One such untested cat species is the tiger (*Panthera tigris*). Once abundant across Asia, tigers are endangered, with fewer than 6,000 individuals remaining in the wild [23]. The Amur tiger subspecies (*Panthera tigris altaica*), present in the Russian Far East and China, has fewer than 550 individuals remaining [23]. Amur tigers are solitary hunters that range widely in search of prey. They prey primarily on wild ungulates such as deer and boar, but will also opportunistically predate on other wild species including badger, raccoon dog, and bear; as well as domestic species such as dog and cow [24–29]. In this study, we sought to identify GHVs in free-ranging Amur tigers of the Russian Far East. In doing so, we identified a GHV in eight Amur tigers that is very closely related to mustelid gammaherpesvirus 1 from badgers. By testing other carnivore species in the local area, we determined that this virus is very common in Asian badgers and likely transmits to Amur tigers via predator-prey interactions. To our knowledge, this is one of the first demonstrations of herpesvirus transmission from prey to predator and highlights the risk that top predators face in exposure to infectious diseases.

## Results

### Identification of a mustelid gammaherpesvirus 1 (MusGHV1) variant in Amur tigers

**Fig 1:**
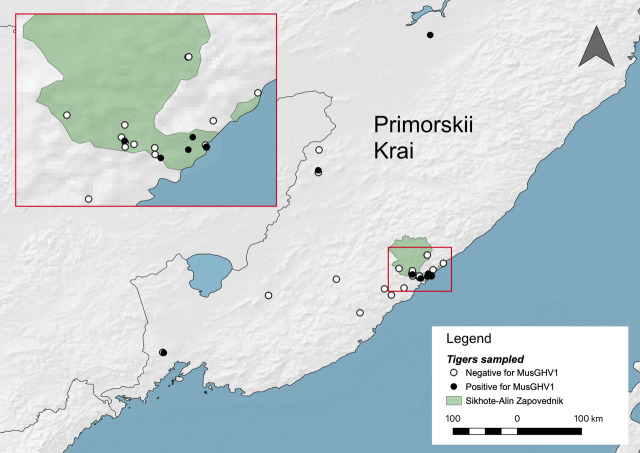
Location of Amur tigers tested for GHVs in the Russian Far East. Open circles indicate tigers that tested negative for GHVs. Filled circles indicate tigers infected with mustelid gammaherpesvirus 1 (MusGHV1). The inset map highlights tigers within and around the Sikhote-Alin Zapovednik (strictly protected area). This map was prepared using Natural Earth (https://www.naturalearthdata.com/), and contains Sikhote-Alin Nature Reserve layer (5475758, version 22) information from OpenStreetMap and OpenStreetMap Foundation, which is made available under the Open Database License (https://www.openstreetmap.org/copyright).

We designed this study to test endangered Amur tigers (*Panthera tigris altaica*) for presence of gammaherpesviruses (GHVs). We extracted DNA from 50 blood samples collected previously from 41 tigers from 1992 to 2014 by the Siberian Tiger Project (a collaborative project started by the Hornocker Wildlife Institute and the Russian Academy of Sciences), and continued by the Wildlife Conservation Society and Sikhote-Alin Biosphere Zapovednik. Samples were collected in and around Sikhote-Alin Biosphere Zapovednik, an IUCN Category I strictly protected nature reserve in the Russian Far East (Fig 1). The presence of intact, amplifiable, and inhibitor-free template DNA in each sample was verified by PCR-amplifying the carnivore glyceraldehyde-3-phosphate dehydrogenase (GAPDH) gene as previously described [17]. We then tested all DNA samples for presence of the conserved glycoprotein B (gB) gene of GHVs using a degenerate, nested PCR designed by Ehlers and colleagues [30] that we and others have previously used to detect and identify dozens of novel GHVs and several betaherpesviruses [13,16–18,30,31]. Three of 41 tigers were PCR-positive for GHV gB gene. Sequences (453 nt) of gB from these three tigers were nearly identical to each other, with the sequence from one tiger having a single synonymous A-to-C polymorphism (S1 Fig). Interestingly, there were three samples available from one positive female tiger over a 2.5-year (30 month) period from April 1995 to October 1997. We found that all three samples from this tiger were gB-positive with nearly identical sequences. The sequence from the final sample had a single nonsynonymous C-to-T mutation that resulted in a Ser-to-Lys amino acid change relative to the other two samples (S1 Fig). Since degenerate PCR is not always highly sensitive for low-level GHV detection, we designed a virus-specific nested PCR. Using this PCR, we re-tested all tiger samples and identified five additional positive tigers for a total of 8/41 positive tigers (19.5% prevalence). gB sequences from the additional tigers were identical to the consensus gB sequence for this GHV, thus GHV gB sequences from all eight positive tigers differed at most by a single nucleotide (>99% identity).

**Fig 2:**
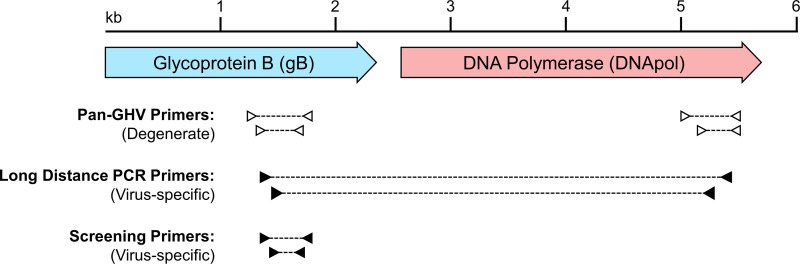
PCR amplification strategy for GHV sequencing. Pan-GHV degenerate primers were used to amplify small sections of glycoprotein B and DNA polymerase genes. Sequences from these regions were then used to design virus-specific primers for amplification and sequencing of an approximately 3.5 kb region spanning these two genes. Additional virus-specific primers targeting glycoprotein B were designed for high-sensitivity screening of samples.

**Fig 3:**
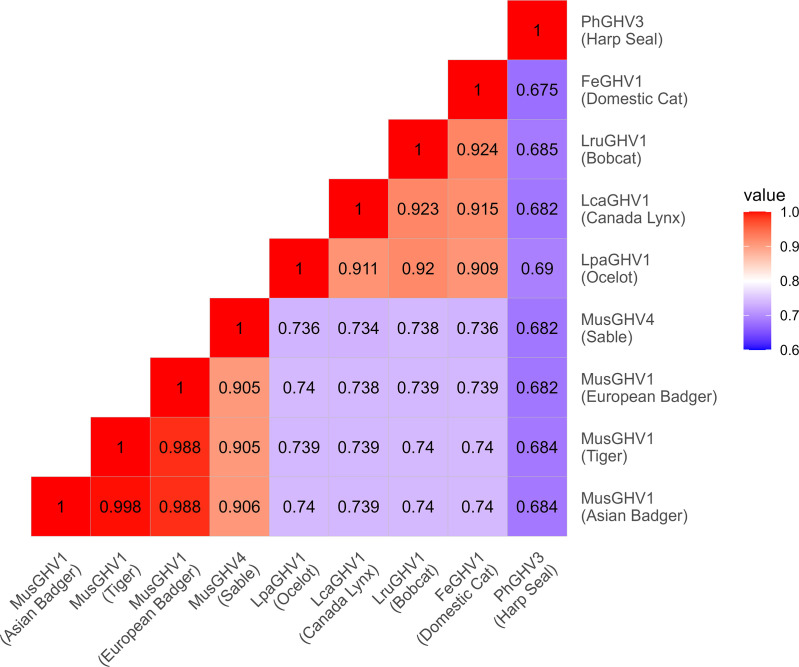
Nucleotide identity matrix for the carnivore host sub-group of GHV genus *Percavirus.* GHV sequences from glycoprotein B through DNA polymerase (approximately 3.5 kb) were aligned using MUSCLE [33]. Nucleotide identity between each pair of sequences is indicated in the matrix.

In order to rigorously compare this GHV in tigers to other known GHVs, we sought to obtain a longer 3.5 kb nucleotide sequence spanning the gB to adjacent DNA polymerase (Dpol) genes. Our group and others have previously used this strategy to compare GHV sequences from diverse hosts [13,17,18]. Using a degenerate PCR targeting a conserved region of the DNA polymerase [32], we amplified and sequenced a short region of Dpol and then used virus-specific primers to amplify and sequence the 3.5 kb gB to Dpol region (Fig 2). Surprisingly, we found that the GHV sequence in tigers had 98.8% nucleotide identity (40 nt differences in 3556 nt; Fig 3) to mustelid gammaherpesvirus 1 (MusGHV1) commonly found in European badgers (*Meles meles*), which do not occur in the Russian Far East. This high level of nucleotide identity suggested that Amur tigers were likely infected with a variant of MusGHV1. Further, this finding suggested that the MusGHV1 variant identified in Amur tigers might not be tiger host-specific, but rather acquired from another animal. MusGHV1 is a member of the *Percavirus* genus of GHVs, and phylogenetically clusters with percaviruses found in hosts from the order Carnivora [17]. Based on this, we hypothesized that the MusGHV1 variant identified in Amur tigers may be found in one or more local carnivore species.

### MusGHV1 variant found in Amur tigers is common in local Asian badgers

To test the hypothesis that the MusGHV1 variant found in Amur tigers was present in other carnivore species, we obtained previously-collected blood samples from animals in the local area. We verified that all DNAs extracted from blood were intact, amplifiable, and inhibitor-free by PCR-amplification of the carnivore GAPDH gene [17]. In total, we tested 69 blood DNA samples from 11 carnivore species for GHVs using degenerate nested PCR for the gB gene (Table 1). This included three species of *Felidae*: *Prionailurus bengalensis* (leopard cat), *Panthera pardus orientalis* (Amur leopard), and *Lynx lynx* (Eurasian lynx); four species of Mustelidae: *Meles leucurus* (Asian badger), *Martes zibellina* (sable), *Mustela siberica* (Siberian weasel), and *Neogale vison* (American mink); two species of Canidae: *Nyctereutes procyonoides* (raccoon dog) and *Vulpes vulpes* (red fox); and two species of Ursidae: *Ursus thibetanus* (Asiatic black bear) and *Ursus arctos* (brown bear). Studies of prey preference have demonstrated that Amur tigers prey on many of these species (Table 1) [24–29]. Five out of 21 Asian badger samples were positive for gB (Table 1). Sequences of 453 nt from the five positive badgers were identical to sequences detected in Amur tigers. We then re-tested badger samples using the virus-specific PCR and found that 17 out of 21 (81.0%) were positive. Sequences of gB from all of these samples were identical to MusGHV1 gB sequences found in Amur tigers (S1 Fig). In contrast, MusGHV1 was not detected in any of the other 10 species of carnivores tested.

**Table 1:** PCR amplification of herpesvirus DNA from carnivore host species.

Family and species	Common name	Tiger prey species?	No. of animals	Year range	Pan-GHV gB pos. animals	Virus-specific PCR positive	Virus identity
**Felidae**							
*Panthera tigris altaica*	Amur tiger	n/a	41	1995–2014	3/41	8/41 (19.5%)	Mustelid GHV1
*Prionailurus bengalensis*	Leopard cat	No	8	2008–2013	0	–	–
*Panthera pardus orientalis*	Amur leopard	No	4	–	0	–	–
*Lynx lynx*	Eurasian lynx	No	1	2010	0	–	–
**Mustelidae**							
*Meles leucurus*	Asian badger	Yes	21	2007–2014	5/21	17/21 (81.0%)	Mustelid GHV1
*Martes zibellina*	Sable	Yes	2	2013	1/2	2/2 (100%)	Mustelid GHV4*
*Mustela sibirica*	Siberian weasel	Potential	2	2006–2007	0	–	–
*Neogale vison*	American mink	Unknown	1	2008	0	–	–
**Canidae**							
*Nyctereutes procyonoides*	Raccoon dog	Yes	11	2007–2014	0	–	–
*Vulpes vulpes*	Red fox	Yes	2	2008–2011	0	–	–
**Ursidae**							
*Ursus thibetanus*	Asiatic black bear	Yes	8	2007–2011	6/8	6/8 (75.0%)	Ursid GHV2*
*Ursus arctos*	Brown bear	Yes	9	1993–2011	4/9	4/9 (44.4%)	Ursid GHV3*

n/a, not applicable

*new virus identified in this study

**Fig 4:**
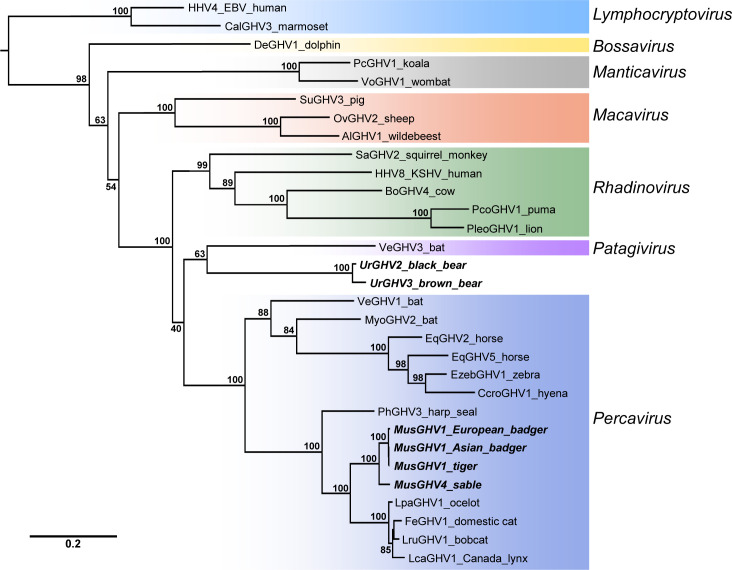
Maximum-likelihood phylogenetic analysis of gammaherpesviruses based on concatenated gB and DNApol amino acid alignments. The betaherpesvirus human cytomegalovirus (HHV5; GenBank accession no. NC006273) was used as an outgroup to root the tree but is not displayed due to space constraints. Bootstrap support based on 1000 replicates is displayed as a percentage out of 100 at each node. Phylogenetic clusters corresponding to established GHV genera are indicated by name and color. Virus abbreviations, their definitions, and NCBI accession numbers are as follows: HHV4, human herpesvirus 4 (Epstein-Barr virus), NC007605; CalGHV3, callitrichine gammaherpesvirus 3, NC004367; DeGHV1, delphinid gammaherpesvirus 1, NC035117; PcGHV1, phascolarctid gammaherpesvirus 1, MG452722; VoGHV1, vombatid gammaherpesvirus 1, MG452721; SuGHV3, suid gammaherpesvirus 3, AF478169; OvGHV2, ovine gammaherpesvirus 2, NC007646; AlGHV1, alcelaphine gammaherpesvirus 1, NC002531; SaGHV2, saimiriine gammaherpesvirus 2, NC001350; HHV8, human herpesvirus 8 (Kaposi’s sarcoma-associated herpesvirus), NC009333; BoGHV4, bovine gammaherpesvirus 4, NC002665; PcoGHV1, *Puma concolor* gammaherpesvirus 1, KF840717; PleoGHV1, *Panthera leo* gammaherpesvirus 1, DQ789370; VeGHV3, vespertilionid gammaherpesvirus 3, MF385016; UrGHV2, ursid gammaherpesvirus 2, MK089801; UrGHV3, ursid gammaherpesvirus 3, OP751953; VeGHV1, vespertilionid gammaherpesvirus 1, KU220026; MyoGHV2, *Myotis ricketti* gammaherpesvirus 2, JN692430; EqGHV2, equid gammaherpesvirus 2, NC001650; EqGHV5, equid gammaherpesvirus 5, NC026421; EzebGHV1, *Equus zebra* gammaherpesvirus 1, AY495965; CcroGHV1, *Crocuta crocuta* gammaherpesvirus 1, DQ789371; PhGHV3, phocid gammaherpesvirus 3, KP136799; MusGHV1, mustelid gammaherpesvirus 1, AF376034, PQ066357, PQ066358; MusGHV4, mustelid gammaherpesvirus 4, OP751954; LpaGHV1, *Leopardus pardalis* gammaherpesvirus 1, KP721220; FeGHV1, felid gammaherpesvirus 1, KT595939; LruGHV1, *Lynx rufus* gammaherpesvirus 1, KF840716; LcaGHV1, *Lynx canadensis* gammaherpesvirus 1, MH520115.

To further characterize MusGHV1 sequence from Asian badgers, we amplified and sequenced the 3.5 kb region spanning gB and Dpol (Fig 2). The long MusGHV1 sequence from Asian badger had 99.8% identity (6 nt differences in 3556 nt) to MusGHV1 from Amur tiger and 98.8% identity (40 nt differences in 3556 nt) to MusGHV1 from European badger (Fig 3). Phylogenetic analyses confirmed clustering of the three MusGHV1 sequences together in all 1000 bootstrap replicates (Fig 4). The MusGHV1 sequences further cluster within a subclade of Percaviruses in which all hosts are carnivores (harp seal, badger, tiger, sable, ocelot, domestic cat, bobcat, and Canada lynx). Collectively, these results indicate that a MusGHV1 variant common in Asian badgers (81.0% prevalence) is also found to infect Amur tigers (19.5% prevalence).

### Location, sex, and age of MusGHV1-infected Amur tigers

We further analyzed whether MusGHV1 infection was associated with tiger location, sex or age. Collection dates for MusGHV1-positive tigers ranged over 16 years from 1995 to 2011. We mapped GIS locations for 8 MusGHV1-positive and 27 negative tigers where GIS information was available (Fig 1). The majority of samples were located in and around the Sikhote-Alin Biosphere Zapovednik. Five of the MusGHV1-positive tigers were located within the Zapovednik. The other three positive tigers were widely dispersed, with the two most distant positive tigers separated by >800 km (Fig 1). MusGHV1-positive tiger locations were generally interspersed with MusGHV1-negative tiger locations. Information on sex was available for 37 of 41 Amur tigers. The prevalence of MusGHV1 was similar among males and females: 23.8% (5/21) in males and 18.8% (3/16) in females. Estimated age of the MusGHV1-positive tigers ranged widely from 1 to 150 months. Median age of MusGHV1-positive tigers was 78 months (interquartile range 15–135 m) compared to 36 months (interquartile range 14.25–84 m) for MusGHV1-negative tigers, but medians were not significantly different (Mann-Whitney test, p = 0.16). Collectively, these data indicate that MusGHV1-positive tigers were widely geographically dispersed rather than clustered and positive tigers did not differ from negative tigers with regard to sex or age.

### Identification of novel GHVs in sable, brown bear, and black bear

Pan-GHV PCR targeting the gB gene yielded a positive result in one of two sable (*Martes zibellina*) (Table 1). Subsequent virus-specific PCR detected an additional positive sable with an identical GHV gB sequence (Table 1). To further characterize GHV in sable, we amplified and sequenced the 3.5 kb region spanning gB and Dpol (Fig 2). Comparison of this sequence to GHV sequences in the NCBI database (www.ncbi.nlm.nih.gov) indicated that the GHV identified in sable had greatest identity to MusGHV1 at 90.6% across the full gB-Dpol region (Fig 3). Phylogenetic analyses clearly indicated that the sable GHV clustered with MusGHV1 (1000/1000 bootstrap replicates) within the Percavirus genus (Fig 4). These results indicate that the GHV identified in sable represents a novel Percavirus species that is distinct from MusGHV1. In accordance with herpesvirus nomenclature, we tentatively assigned this new virus the name mustelid gammaherpesvirus 4 (MusGHV4) as the fourth GHV identified in mustelids.

Pan-GHV PCR targeting the gB gene also produced positive results in 6 of 8 Asiatic black bears (*Ursus thibetanus*), and 4 of 9 brown bears (*Ursus arctos*) (Table 1). The 6 black bear GHV sequences were identical to each other except for a single synonymous C-to-A substitution in 2/6 sequences (S2 Fig). The 4 brown bear GHV sequences were identical to each other, but distinct from the black bear GHV sequences (93.6% nt identity in gB; 29 differences in 453 nt; S2 Fig). Virus-specific PCR gave results identical to the pan-GHV gB PCR (Table 1). To further characterize the bear GHVs, we amplified and sequenced the 3.5 kb region spanning gB and Dpol for representative samples from both species (Fig 2). The black bear and brown bear GHVs had 91.0% nucleotide identity to each other across a 3682 nt gB-Dpol region. Comparison to sequences in the NCBI database further indicated similarity to Ursid gammaherpesvirus 1 (UrsGHV1) previously identified in captive sun bears (*Helarctos malayanus*) [34]. The black bear and brown bear GHVs had 94.8% and 93.8% identity, respectively, to UrsGHV1 across a 1339 nt region of Dpol (gB sequence not available for UrsGHV1). Phylogenetic analyses of the bear GHVs indicated that they clustered together (1000/1000 bootstrap replicates) and were highly divergent from other GHVs (Fig 4). UrsGHV1 was not included in the phylogeny because the full gB-Dpol sequence was not available. While the bear GHV sequences did not cluster closely with any other GHVs, the phylogeny indicated a potential distant relationship with moderate bootstrap support (630/1000 replicates) to vespertilionid gammaherpesvirus 3 of big brown bats (*Eptesicus fuscus*), separated by long branches indicative of large genetic distance (Fig 4). These results indicate two distinct and novel GHV species, one found in black bears and a second found in brown bears. In accordance with herpesvirus nomenclature, we tentatively assigned the black bear GHV as Ursid gammaherpesvirus 2 (UrsGHV2) and the brown bear GHV as Ursid gammaherpesvirus 3 (UrsGHV3). With further genetic characterization, UrsGHVs 1–3 may warrant creation of a new GHV genus.

## Discussion

In this study, we identified a mustelid gammaherpesvirus 1 (MusGHV1) variant present in 8 of 41 (19.5%) Amur tigers located over a wide geographic area in the Russian Far East. Since GHVs typically establish life-long infection of lymphoid cells, the presence of MusGHV1 DNA in Amur tiger blood samples is consistent with persistent virus infection rather than transient exposure. Further support for persistent infection was indicated by our finding that one Amur tiger was MusGHV1-positive in 3 sequential blood samples collected over a 2.5-year period. Upon screening of 11 local carnivore species for GHVs, we found the same variant MusGHV1 present in 17 of 21 (81.0%) Asian badgers, but not in 10 other carnivore species. The high prevalence of this MusGHV1 variant in Asian badgers suggests that they are likely the primary host species for the virus. This is further supported by genetic similarity to the well-characterized and highly prevalent MusGHV1 of European badgers [20,35]. Studies of Amur tiger prey preference based on analysis of tiger scat clearly indicate that Asian badgers are a minor, but very common part of the Amur tiger diet in the Russian Far East [24,26–29]. Likewise, in a study of GPS-collared Amur tiger kill sites, 6.5% of kill sites were badgers, the greatest percentage for any species other than the deer and boar species that make up the bulk of the Amur tiger diet [25]. Thus, it appears likely that Amur tigers became infected with MusGHV1 through predator-prey interaction with Asian badgers. While these results strongly support tiger predation of badgers as the likely means of MusGHV1 spillover, we considered other possibilities. Could tigers contact MusGHV1 shed in the environment by badgers or another animal? In GHV infections, viral latency predominates, with generally infrequent reactivation and shedding [1,36]. Infrequent shedding, especially in the vast landscape of the Russian Far East, makes environmental transmission exceedingly unlikely. Could MusGHV1 be transmitted via contaminated fomites, such as carcasses scavenged by both tigers and badgers? While this transmission route might be possible, badgers are relatively rare scavengers [37,38], making this a low-frequency event also likely to deposit a low amount of virus. Could there be a third infected species eaten by both predators? This possibility is also relatively unlikely as there is not an obvious species they both prey on, with no clear overlap between the diets of Amur tigers [24–29] and Asian badgers [39–41]. With regard to potential involvement of other carnivores, we tested the most numerous carnivore species present in the local ecosystem for GHVs, but we could not feasibly test every local animal species, so there is an unlikely possibility that another untested species could also be infected with MusGHV1 and play a role in transmission to Amur tigers. Overall, the high prevalence of MusGHV1 in Asian badgers (81.0%) and the fact that badgers make up a common part of the Amur tiger diet strongly support predator-prey interactions as the most likely route of MusGHV1 spillover from badgers to tigers. Once Amur tigers become infected, it is unknown whether they can transmit MusGHV1 to other Amur tigers. The solitary nature of this species suggests that potential opportunities for transmission might be limited to mating and maternal care for cubs. Interestingly, one of the MusGHV1-positive tigers in this study was a juvenile found newly dead and estimated to be one month old. Juveniles of this age typically are still being nursed and not consuming meat, suggesting the possibility of mother-to-cub transmission.

Transmission of pathogens from prey to predator is a well-described phenomenon that presents unique risk for top predators [14]. However, for the highly host species-specific GHVs, there have been no clearly-documented cases of predator-prey transmission, to our knowledge. A study set up specifically to look for virus transmission from prey primates to predator primates detected no GHV transmission [15] but retrovirus transmission from prey-to-predator in the same animals was detected frequently [42], suggesting GHV-specific host barriers to transmission. Likewise, while Amur tigers prey on sable and bears [24–29], our study found that GHVs common in these prey species were not detected in tigers. However, there have been hints that predator-prey and carnivore-to-carnivore transmission of GHVs may occur. The close phylogenetic relationship between zebra and hyena GHVs suggests a potential ancient predator-prey transmission origin for hyena GHV [13]. Furthermore, we and others have documented individual GHVs found in two or more different felid species [17,18,21], suggesting that GHVs may be transmitted between carnivore species or possibly transmitted from a common unknown prey species. Our finding of MusGHV1 transmission from Asian badgers to Amur tigers provides the first strongly-supported example of GHV predator-prey transmission, to our knowledge. The particular virus and host characteristics necessary to facilitate successful cross-species transmission in this case are not clear. Evolutionary adaptation of GHVs to particular host species likely limits their potential transmission to divergent hosts. In contrast, cross-species GHV transmission between closely-related species may present fewer intrinsic host barriers [43–45]. Thus, top predator carnivores such as the Amur tiger may be at special risk of cross-species infection by preying on related species from the order *Carnivora*, such as the Asian badger, as opposed to preying on infected non-carnivores. Furthermore, virus-specific factors may play a role in facilitating or prohibiting cross-species transmission. In this context, it is notable that mustelid and felid GHVs form closely-related “sister” phylogenetic clades within the carnivoran percaviruses (Fig 4), suggesting that the properties of a GHV necessary to infect a felid are likely to be relatively well-conserved in a mustelid GHV. In contrast, the potential risk of MusGHV1 spillover to wild or domestic non-carnivore hosts is likely low.

Amur tigers in the Russian Far East and China are highly threatened, with less than 550 individuals remaining in the wild [23]. Key threats to Amur tiger conservation include poaching, loss of prey species, and infectious diseases. Among infectious disease threats, canine distemper virus is particularly important due to its high virulence in tigers and sporadic transmission from a wild carnivore reservoir and possibly domestic dogs [46]. In contrast, it is unlikely that MusGHV1 causes acute virulent disease in Amur tigers based on the generally low pathogenicity of most GHVs in immune-competent animals. However, it is unknown whether MusGHV1 might have more subtle pathogenic consequences. Interestingly, a recent series of studies by Tsai et al. found that MusGHV1 reactivation in the European badger genital tract was associated with stress response [36] and *Clostridium* bacterial infection [47] and negatively correlated with reproductive success [9]. These findings suggest that MusGHV1 may impact badger health and fitness and highlight a plausible means by which MusGHV1 infection could similarly impact tiger fitness. In addition, the well-characterized association of GHVs with lymphoproliferative diseases in humans and animals [3–6,48] suggests another potential health impact of MusGHV1 infection for investigation. Future studies of Amur tiger health should consider MusGHV1 infection as a parameter.

In this study, testing of local carnivores of the Russian Far East in addition to Amur tigers and Asian badgers revealed three novel GHVs, each found in a single host species: MusGHV4 in sable, UrsGHV2 in black bear, and UrsGHV3 in brown bear. MusGHV4 is closely related to MusGHV1, with 90.6% nucleotide identity (Fig 3) and strongly-supported phylogenetic clustering (Fig 4). Additional short GHV sequences from a conserved part of Dpol are available for several other mustelids, including fisher [49], American marten [50], Northern sea otter [51], and Eurasian otter (MT266854). These sequences have relatively high nucleotide identity (>85%) to MusGHV1 and MusGHV4, suggesting that there is likely a larger sub-clade of related mustelid percaviruses that cluster separately from felid percaviruses (Fig 4). The bear GHVs, UrsGHV2 and UrsGHV3, were closely related to each other with 91.0% nucleotide identity and strongly-supported phylogenetic clustering (Fig 4). These viruses are highly unique in that they do not cluster phylogenetically with established GHV genera (Fig 4) and thus may ultimately warrant creation of a new GHV genus following future complete genome sequencing. Our phylogenetic analysis revealed a potential distant relationship to VeGHV3 of bats (Fig 4). This result may lend support to the previous observation that bat GHV sequences tend to be positioned basally to clusters of other mammalian GHVs, suggesting that bat GHVs may have been progenitors of GHVs now present in other mammals [11]. However, the relationship between bear GHVs and VeGHV3 should be interpreted with caution due to low bootstrap support (630/1000) and long branches separating these GHVs (Fig 4). The bear GHVs were found to be highly host species-specific in that only black bears had UrsGHV2 and only brown bears had UrsGHV3, despite these species being located in the same geographic area. This result is typical of the generally high host species-specificity of many GHVs and stands in contrast to our findings for MusGHV1. Future investigation of these novel ursid GHVs with regard to pathology and disease association in bears is warranted as related ursid GHVs have been identified in tentative association with carcinoma and neurologic disease [34,52,53].

In this study, we found that a MusGHV1 variant common in Asian badgers can cross species barriers to infect endangered Amur tigers of the Russian Far East, with transmission very likely occurring via predator-prey interaction. This is one of the first examples, to our knowledge, of GHV transmission from prey to predator. This finding provides support for the idea that top predators are at particular risk for cross-species infection with pathogens. In particular, carnivoran predators that prey on fellow members of the order *Carnivora* may be uniquely susceptible to cross-species infection with host-adapted viruses such as GHVs.

## Materials and methods

### Sample collection and DNA preparation

Blood samples from tigers and other wild carnivores were obtained from the archives of the Wildlife Conservation Society (New York, NY, USA) and were collected from wild carnivores captured for research purposes or in response to incidents of human-carnivore conflict. All procedures involving handling of free-ranging animals were approved by the Wildlife Conservation Society’s Institutional Animal Care and Use Committee. Tiger samples were collected in Primorskii Krai (38 animals), neighboring Khabarovskii Krai (two animals), and Amurskaya Oblast (one animal) between 1992 and 2014. Samples from other carnivores were collected in the same regions between 1993 and 2014 as part of on-going ecological studies, or as part of epidemiological research focused on canine distemper virus. Animals were captured using cage traps or Aldrich foot snares [54], appropriate techniques for most of the 17 terrestrial carnivore species that occur in the Russian Far East. Wildlife blood samples were collected in EDTA tubes and stored at −20°C for up to 8 y prior to export, after which they were transferred to −80°C and shipped using dry ice for analysis. Total DNA was extracted from thawed whole blood or purified white blood cells using a DNeasy blood and tissue kit (Qiagen, Valencia, CA, USA). DNA concentration was determined using a NanoDrop 1000 UV-Vis spectrophotometer (NanoDrop Technologies, Wilmington, DE, USA) and only samples with ≥5 ng/µl DNA were included for analysis. The presence of intact, amplifiable, and inhibitor-free template DNA in each sample was verified by PCR-amplifying the carnivore glyceraldehyde-3-phosphate dehydrogenase (GAPDH) gene as previously described [17].

### Amplification of GHV Glycoprotein B (gB) Sequence Using Degenerate Primers

Pan-GHV amplification of gB gene was conducted using a degenerate nested PCR with primer set GH1 previously described by Ehlers et al. [13]. In the first round, 50–500 ng DNA was added to 50 µl reaction mixtures containing 2 units Platinum *Taq* polymerase (ThermoFisher, Carlsbad, CA), 1 µM primers 2759s and 2762as, 1.5 mM MgCl_2_, 0.2 mM deoxynucleoside triphosphates, and 1X PCR buffer (ThermoFisher). Cycling conditions included an initial denaturation step of 94°C for 2 min; followed by 45 cycles of 94°C for 30 s, 46°C for 30 s, and 72°C for 30 s; and a final extension at 72°C for 7 min. In the second round, 2 µl of first round reaction product was used as the template and reactions were conducted under conditions identical to the first round, except using primers 2760s and 2761as. PCR products of ~500 bp were purified with a QIAquick PCR purification kit (Qiagen) and Sanger sequenced in both directions.

### GHV-Specific PCRs.

MusGHV1 and MusGHV4 were detected by PCR using gB primers PCV-F1 and PCV-R1, designed to be specific for GHVs of the carnivore sub-lineage of Percaviruses (Table 2). UrsGHV2 and UrsGHV3 were detected using Ursid GHV-specific gB primers BGHV-F1 and BGHV-R1 (Table 2). For both GHV-specific PCRs, template DNA (50–500 ng) was added to 50 µl reaction mixtures containing 1 unit Platinum *Taq* polymerase (ThermoFisher), 200 nM primers, 1.5 mM MgCl_2_, 0.2 mM deoxynucleoside triphosphates, and 1X PCR buffer (ThermoFisher). Cycling conditions for reactions with PCV primers included an initial denaturation step of 94°C for 2 min; followed by 45 cycles of 94°C for 30 s, 57°C for 30 s, and 72°C for 30 s; and a final extension at 72°C for 7 min. Cycling conditions for reactions with BGHV primers were identical, except an annealing temperature of 60°C was used. PCR products of expected size (281 bp for PCV primers, 224 bp for BGHV primers) were purified using a QIAquick PCR purification kit (Qiagen), and Sanger sequenced in both directions.

**Table 2:** PCR primers designed in this study.

Primer Set	Primer Name	Use^1^	Sequence (5’ → 3’)
Percavirus-specific	PCV-F1	Forward	TGTAGACCAAACAAAAGTTGACC
	PCV-R1	Reverse	TGTTGAGGGTAGAAAAATTGTGA
Ursid GHV-specific	BGHV-F1	Forward	CCCGTGTCAGCGAAGATACT
	BGHV-R1	Reverse	GCCTCCACCAAACTGGTCAT
MusGHV1 long-dist	TGHV-F1	1st for	GCAATCTATGGGAAGCCAGT
	TGHV-R3	1st rev	TGACCCTAAAGTTGGCATCATA
	TGHV-F2	2nd for	TGACCTTCACCAAAGCATGAG
	TGHV-R4	2nd rev	GGTTTGTGTATAATTCTTTCGAGTTCA
MusGHV4 long-dist	SBL-F1	1st for	GCGATCTATGGGAAGCCAGT
	SBL-R3	1st rev	TAACCCTAAAGCAGGCATCATA
	SBL-F2	2nd for	TGACCTGCATCAAAGCATGAG
	SBL-R4	2nd rev	TTATTGGTCTGTGTATGATTCTCTCG
Ursid GHV long-dist	BGHV-F2	1st for	AGCGAAGATACTTGGGGATG
	BGHV-R2	1st rev	CTCTTCTTGTTATGACCGAGAGC
	BGHV-F3	2nd for	GTGTTCAGGGGACAGTTGG
	BGHV-R3	2nd rev	GCCGTGATTGCCTCAATG

^1^Forward (for) or reverse (rev) primers and first (1st) or second (2nd) round of PCR.

### Amplification of GHV DNA polymerase (Dpol) by degenerate PCR

Individual GHV species required different procedures for amplification of the Dpol gene. Dpol from bear GHVs (UrsGHV2 and UrsGHV3) was amplified using previously published heminested primers: DFASA and GDTD1B in the first round and VYGA and GDTD1B in the second round [32]. The reaction mix was identical to that for gB degenerate PCR and cycling conditions for both rounds included an initial denaturation step of 94°C for 2 min; followed by 35 cycles of 94°C for 1 min, 60°C for 1 min, and 72°C for 1 min; and a final extension at 72°C for 7 min. Dpol from mustelid GHVs (MusGHV1 and MusGHV4) was amplified using previously published heminested primers: LSSG and GDTD2 in the first round and VYGA2 and GDTD2 in the second round [18]. PCR conditions were identical to those used to amplify Dpol from bear GHVs except that 45 cycles and an annealing temperature of 55°C were used. PCR products of expected size (200–250 bp) were purified using a QIAquick PCR purification kit (Qiagen), and Sanger sequenced in both directions.

### Long-distance PCR

For each GHV, virus-specific nested forward and reverse primers were designed for each GHV based on short gB and Dpol sequences, respectively (Table 2). These primers were used to PCR amplify the ~ 3.5 kb gB to Dpol region for sequencing. In the first round, 50–500 ng DNA was added to 50 µl reaction mixtures containing 2 units High Fidelity Platinum *Taq* polymerase (ThermoFisher), 400 nM primers (Table 2), 2 mM MgSO_4_, 0.2 mM deoxynucleoside triphosphates, and 1X High Fidelity Platinum Taq PCR buffer (ThermoFisher). Cycling conditions included an initial denaturation step of 94°C for 2 min; followed by 40 cycles of 94°C for 30 s, 57°C for 30 s, and 68°C for 4 min; and a final extension at 68°C for 7 min. In the second round, 2 µl of first round reaction product was used as the template and reactions were conducted under conditions identical to the first round, except using the listed second round primers (Table 2). PCR products of ~3.5 kb were purified with a QIAquick PCR purification kit (Qiagen) and Sanger sequenced by primer walking.

### Phylogenetic analysis

Dpol and gB amino acid sequences derived from partial gene sequences were aligned using T-Coffee with default parameters [55]. All positions with gaps and areas where the alignment was poorly supported were manually removed. The resulting Dpol and gB alignments were concatenated to form a single amino acid alignment with 927 positions for phylogenetic analysis. A maximum likelihood phylogenetic analysis was performed using IQ-TREE v2.0 [56] with automatic model selection. The best-fit model of amino acid substitution was the model of Le and Gascuel [57] with invariant sites and gamma distribution with four discrete categories. Bootstrap analyses were performed with 1000 iterations to evaluate the support for each node. The betaherpesvirus human cytomegalovirus was used as an outgroup to root the tree.

### Nucleotide sequence accession numbers

Nucleotide sequences determined in this study were deposited into the NCBI sequence database (https://www.ncbi.nlm.nih.gov/) under the following accession numbers: mustelid gammaherpesvirus 1 from Asian badger, PQ066357; mustelid gammaherpesvirus 1 from Amur tiger, PQ066358; mustelid gammaherpesvirus 4 from sable, OP751954; ursid gammaherpesvirus 2 from black bear, MK089801; and ursid gammaherpesvirus 3 from brown bear, OP751953.

## Supporting information

S1 Fig Alignment of mustelid gammaherpesvirus 1 (MusGHV1) glycoprotein B sequences determined in this study. Each sequence shown was determined in the number of samples indicated in parentheses (n = #). Dots in the alignment indicate nucleotides that are identical to the reference sequence. Dashes indicate regions that were not sequenced due to use of a virus-specific PCR resulting in shorter length amplicons.
(PDF)

S2 Fig Alignment of Ursid GHV glycoprotein B sequences determined in this study. Each sequence shown was determined in the number of samples indicated in parentheses (n = #). Dots in the alignment indicate nucleotides that are identical to the reference sequence.
(PDF)
