# Supplementary material for: Predator-prey transmission of a gammaherpesvirus from Asian badgers (Meles leucurus) to endangered Amur tigers (Panthera tigris altaica)
Source: PLoS One. 2025 Jul 2;20(7):e0327463. doi: 10.1371/journal.pone.0327463 (PMC12221012; doi:10.1371/journal.pone.0327463)
Supplement: S1 Fig — Each sequence shown was determined in the number of samples indicated in parentheses (n = #). Dots in the alignment indicate nucleotides that are identical to the reference sequence. Dashes indicate regions that were not sequenced due to use of a virus-specific PCR resulting in shorter length amplicons. (PDF) [file pone.0327463.s001.pdf]

```
MusGHV1 Asian badger (n=5)  AGTGCAATCTATGGGAAGCCAGTCTCGGCTAGGTTTTTGGGGGATGTCATTTCGGTAACTGAATGTGTGATTGTAGACCAAACAAAAGTTGACCTTCACC  100
MusGHV1 Asian badger (n=12) ----------------------------------------------------------------------------------------------------  100
MusGHV1 Tiger        (n=3)  ....................................................................................................  100
MusGHV1 Tiger        (n=5)  ----------------------------------------------------------------------------------------------------  100
MusGHV1 Tiger        (n=1)  ....................................................................................................  100
MusGHV1 Tiger        (n=1)  ........................................................C...........................................  100

MusGHV1 Asian badger (n=5)  AAAGCATGAGAGTACCAGGCCCTGATAATGTGTGTTATTCTAGACCCATTGTAACTTTCAAATTTAAAAATGGTACTGATGTCTTCACTGGACAATTGGG  200
MusGHV1 Asian badger (n=12) -------------------------...........................................................................  200
MusGHV1 Tiger        (n=3)  ....................................................................................................  200
MusGHV1 Tiger        (n=5)  -------------------------...........................................................................  200
MusGHV1 Tiger        (n=1)  ....................................................................................................  200
MusGHV1 Tiger        (n=1)  ....................................................................................................  200

MusGHV1 Asian badger (n=5)  CCCCCGAAATGAAATCCTCCTATCAACTAATTTGGTTGAGACTTGCAAAGACTCTGCTGTACACTATTTCCAATCTGGTCATCAGATGCACAAGTTTGTC  300
MusGHV1 Asian badger (n=12) ....................................................................................................  300
MusGHV1 Tiger        (n=3)  ....................................................................................................  300
MusGHV1 Tiger        (n=5)  ....................................................................................................  300
MusGHV1 Tiger        (n=1)  .......................T............................................................................  300
MusGHV1 Tiger        (n=1)  ....................................................................................................  300

MusGHV1 Asian badger (n=5)  AATTACCATCATCACAGCACTATAGATATTCACAATTTTTCTACCCTCAACACCTTTATGGCCTTGAACCTAACATTTATTGAAAATATAGACTTTGAGG  400
MusGHV1 Asian badger (n=12) .............................-----------------------------------------------------------------------  400
MusGHV1 Tiger        (n=3)  ....................................................................................................  400
MusGHV1 Tiger        (n=5)  .............................-----------------------------------------------------------------------  400
MusGHV1 Tiger        (n=1)  ....................................................................................................  400
MusGHV1 Tiger        (n=1)  ....................................................................................................  400

MusGHV1 Asian badger (n=5)  TGGTTGAACTCTACTCTAAAGAGGAAAAAAGACTAGCTAATGTTTTAGATATA  453
MusGHV1 Asian badger (n=12) -----------------------------------------------------  453
MusGHV1 Tiger        (n=3)  .....................................................  453
MusGHV1 Tiger        (n=5)  -----------------------------------------------------  453
MusGHV1 Tiger        (n=1)  .....................................................  453
MusGHV1 Tiger        (n=1)  .....................................................  453
```

S1 Fig. Alignment of mustelid gammaherpesvirus 1 (MusGHV1) glycoprotein B sequences determined in this study. Each sequence shown was determined in the number of samples indicated in parentheses (n=#). Dots in the alignment indicate nucleotides that are identical to the reference sequence. Dashes indicate regions that were not sequenced due to use of a virus-specific PCR resulting in shorter length amplicons.
