# Supplementary material for: Predator-prey transmission of a gammaherpesvirus from Asian badgers (Meles leucurus) to endangered Amur tigers (Panthera tigris altaica)
Source: PLoS One. 2025 Jul 2;20(7):e0327463. doi: 10.1371/journal.pone.0327463 (PMC12221012; doi:10.1371/journal.pone.0327463)
Supplement: S2 Fig — Each sequence shown was determined in the number of samples indicated in parentheses (n = #). Dots in the alignment indicate nucleotides that are identical to the reference sequence. (PDF) [file pone.0327463.s002.pdf]

```
UrsGHV3 Brown_bear (n=4)  TCTGCGATCTACGGGAAACCCGTGTCAGCGAAGATACTTGGGGATGTCATCTCCGTCACGCAGTGCATTATAGTTGACCAGAGCAGCGTGTCTGTGCATA  100
UrsGHV2 Black_bear (n=4)  ..G..........................................................................T.....T........G.......  100
UrsGHV2 Black_bear (n=2)  ..G...............................................A..........................T.....T........G.......  100

UrsGHV3 Brown_bear (n=4)  AGAGCCTGAGAATCCCCGATAATCCCACAGTTTGTTACTCGAGACCGCCAGTGACCTTTAAATTTGTAAATGGAACCACTGTGTTCAGGGGACAGTTGGG  200
UrsGHV2 Black_bear (n=4)  .............A..............C....................G.............................C....................  200
UrsGHV2 Black_bear (n=2)  .............A..............C....................G.............................C....................  200

UrsGHV3 Brown_bear (n=4)  ACCCAGGAATGAGATCCTGCTCATGACCAGTTTGGTGGAGGCGTGTCAAGATTCAACGGAATATTACTTTCAAGCGGGAAATGAGATACACCTGTACAAA  300
UrsGHV2 Black_bear (n=4)  G..A......................................A.....G..........................A........................  300
UrsGHV2 Black_bear (n=2)  G..A......................................A.....G..........................A........................  300

UrsGHV3 Brown_bear (n=4)  GACTACGTGCACAAAGATACGATAGCCATCTCAAACATCACTACTCTTGATACCTTCATAGCATTGAATATCTCCTTCATAGAGAATATAGACTTTCAAG  400
UrsGHV2 Black_bear (n=4)  ........T........C..A......G.............C.................T..CC....C...............................  400
UrsGHV2 Black_bear (n=2)  ........T........C..A......G.............C.................T..CC....C...............................  400

UrsGHV3 Brown_bear (n=4)  TGATAGAATTATATTCCAAAAATGAAAAACATCTAGCCAATGTTTTTGACCTG  453
UrsGHV2 Black_bear (n=4)  .............C..T...........G.....G..A..C...........A  453
UrsGHV2 Black_bear (n=2)  .............C..T...........G.....G..A..C...........A  453
```

S2 Fig. Alignment of Ursid GHV glycoprotein B sequences determined in this study. Each sequence shown was determined in the number of samples indicated in parentheses (n=#). Dots in the alignment indicate nucleotides that are identical to the reference sequence.
